# Optimizing a Human Papillomavirus Type 16 L1-Based Chimaeric Gene for Expression in Plants

**DOI:** 10.3389/fbioe.2018.00101

**Published:** 2018-07-16

**Authors:** Inga I. Hitzeroth, Aleyo Chabeda, Mark P. Whitehead, Marcus Graf, Edward P. Rybicki

**Affiliations:** ^1^Biopharming Research Unit, Department of Molecular and Cell Biology, University of Cape Town, Rondebosch, South Africa; ^2^Thermo Fisher Scientific GENEART GmbH, Regensburg, Germany; ^3^Faculty of Health Sciences, Institute of Infectious Disease and Molecular Medicine, University of Cape Town, Observatory, South Africa

**Keywords:** HPV-16, L1/L2chimera, plant expression, codon usage, L1, regulatory elements

## Abstract

Human papillomaviruses (HPVs) are the causative agents of cervical cancer, the fourth most prevalent cancer in women worldwide. The major capsid protein L1 self-assembles into virus-like particles (VLPs), even in the absence of the minor L2 protein: such VLPs have successfully been used as prophylactic vaccines. There remains a need, however, to develop cheaper vaccines that protect against a wider range of HPV types. The use of all or parts of the L2 minor capsid protein can potentially address this issue, as it has sequence regions conserved across several HPV types, which can elicit a wider spectrum of cross-neutralizing antibodies. Production of HPV VLPs in plants is a viable option to reduce costs; the use of a L1/L2 chimera which has previously elicited a cross-protective immune response is an option to broaden cross-protection. The objective of this study was to investigate the effect of codon optimization and of increasing the G+C content of synthetic L1/L2 genes on protein expression in plants. Additionally, we replaced varying portions of the 5′ region of the *L1* gene with the wild type (*wt*) viral sequence to determine the effect of several negative regulatory elements on expression. We showed that GC-rich genes resulted in a 10-fold increase of mRNA levels and 3-fold higher accumulation of proteins. However, the highest increase of expression was achieved with a high GC-content human codon-optimized gene, which resulted in a 100-fold increase in mRNA levels and 8- to 9-fold increase in protein levels. Changing the 5′ end of the *L1* gene back to its *wt* sequence decreased mRNA and protein expression. Our results suggest that the negative elements in the 5′ end of *L1* are inadvertently destroyed by changing the codon usage, which enhances protein expression.

## Introduction

Cervical cancer is the fourth most common cancer among women worldwide (Ferlay et al., 2015), and the causal association between Human papillomavirus (HPV) infection and cervical cancer has been well described (zur Hausen et al., 1981; zur Hausen, 1996). Three multivalent HPV prophylactic vaccines based on L1 virus-like proteins (VLPs) have been licensed, and are highly effective in the prevention of vaccine-type infections and associated disease (Schiller et al., 2008; Joura et al., 2015; Toh et al., 2015; Signorelli et al., 2017). Cervarix™ (GlaxoSmithKline) contains L1 VLPs from types 16 and 18; Gardasil® (Merck & Co., Inc.) contains L1 VLPs of low-risk genital wart types 6 and 11 and high cancer-risk types 16 and 18; and Gardasil® 9 (Merck & Co., Inc.), the most recently approved nonavalent vaccine, targets HPV-6/11/16/18, and an additional five HPV types (HPV-31/33/45/52/58). Despite the success of these vaccines, the cervical cancer burden remains high. Additionally, these vaccines are expensive and show type-restrictive prophylactic efficacy.

To address the need for vaccines that will protect against more than one HPV type, the L2 minor capsid protein has been investigated as a vaccine candidate (Karanam et al., 2009). Sequences within the HPV-16 L2 N-terminal region, especially amino acids (aa) 1–120, contain broadly cross-neutralizing epitopes that can neutralize a broad range of mucosal and cutaneous HPVs (Pastrana et al., 2005; Alphs et al., 2008). However, in the context of the native virion this L2 sequence is not prominently displayed: while residues 1–88 are accessible to antibodies during infection, there are only 12–36 molecules per natural virion (Guan et al., 2017). Additionally, although the L2 N-terminal region contains broadly cross-neutralizing epitopes, L2 is also subdominant to L1 as an antigen, and the use of L1+L2 VLPs in vaccination does not confer more cross-protection in animals compared to use of L1 VLPs only (Roden et al., 2000). Accordingly, increasing the density of these sequences at the surface of the particles in a different context than the immunodominent L1 pentamer would almost certainly significantly increase their immunogenicity.

The HPV-16 L2 residues 108–120 (LVEETSFIDAGAP; L2_108−−120_) form a common neutralizing epitope across HPV-16, − 6, − 11, and − 18 (Kawana et al., 2001). We have previously used this epitope in a number of chimaeric constructs for the production of a potential multivalent vaccine candidate, with the L2_108−120_ replacing L1 residues in a number of selected surface-exposed regions that do not impact L1 conformational epitopes, in both insect cells and in plants (Varsani et al., 2003a; McGrath et al., 2013; Pineo et al., 2013). A consistent finding was that replacement of part of the h4 helix with the L2 peptide resulted in the best immunogenicity.

Plant expression systems present a cost-effective alternative to conventional vaccine production due to their scalability, rapid production and low risk of contamination (Fischer et al., 2004; Rybicki, 2010; Merlin et al., 2014). Estimates suggest that generic production cost of goods could be reduced by 50% compared to conventional production (Nandi et al., 2016). Various HPV proteins have been expressed in plants (reviewed in Rybicki, 2014). Plant-derived HPV L1 proteins self-assemble into higher-order structures, and are both immunogenic and show protective efficacy as vaccines in animal models (Kohl et al., 2006). Transient plant expression systems are particularly useful for the rapid production of antigens (Rybicki, 2010), and significantly higher L1 protein levels have been obtained in comparison to stable nuclear transformation (Varsani et al., 2003b; Giorgi et al., 2010). Low yields of recombinant protein have often been reported using plant expression systems; however, methods such as the use of strong plant promoters (Twyman et al., 2003; Obembe et al., 2011), codon optimization (Biemelt et al., 2003; Maclean et al., 2007), co-expression of silencing suppressors (Takeda et al., 2002; Voinnet et al., 2003), and subcellular targeting have been used to increase protein yields (Maclean et al., 2007; Twyman et al., 2013).

Our previous investigation of HPV-16 L1 transient expression in plants determined that use of a human codon-optimized gene, and targeting protein accumulation to the chloroplast rather than the cytosol, ER, or apoplast, resulted in highest accumulation of HPV-16 L1 protein; in contrast, a plant codon-optimized gene was not expressed at meaningful levels (Maclean et al., 2007). Similar results for independently made plant and human codon-optimized HPV-16 L1 genes were obtained by Biemelt et al. (2003). Given the counter-intuitive results in both investigations, it is clear that further insights into optimization of protein expression are necessary.

In our previous work, the *wt* HPV-16 *L1* had a GC content of 38% and the plant codon-optimized gene 35%, whereas the human codon-optimized *L1* (*hL1*) had a GC content of 63%. This led us to hypothesize that expression levels in plants are determined at least in part by GC content. This is similar to the situation for mammalian cells, where GC-rich genes are better expressed than AT-rich genes (Kudla et al., 2006). Another factor affecting mammalian cell expression of HPV L1 genes specifically are negative control elements acting at the transcriptional level (Collier et al., 2002; Rollman et al., 2004; Johansson and Schwartz, 2013). It is thought that these negative elements control L1 expression during epithelial cell differentiation, as it is only produced at later stages of the virus life cycle and at a late stage in differentiation. Codon optimization changes the sequence of these elements and thereby significantly impacts their functions.

We report here on the use of *Agrobacterium*-mediated transient expression in *Nicotiana benthamiana* of seven HPV-16-derived genes—six synthetic and one *wt* L1/L2_108−120_–encoding the single L1/L2 chimera of interest as a candidate vaccine, in order to investigate the impact of codon alteration and overall GC content on the accumulation of the protein. We investigated if differences in expression were at the transcriptional level, as well as exploring whether destruction of known negative regulatory elements are involved in determining protein expression level, by replacing parts of the 5′ region of the L1/L2 chimera gene with *wt L1* DNA sequence.

## Methods

### Synthesis of the *L1/L2* and *wt/L1/L2* chimeras

**Figure 1:**
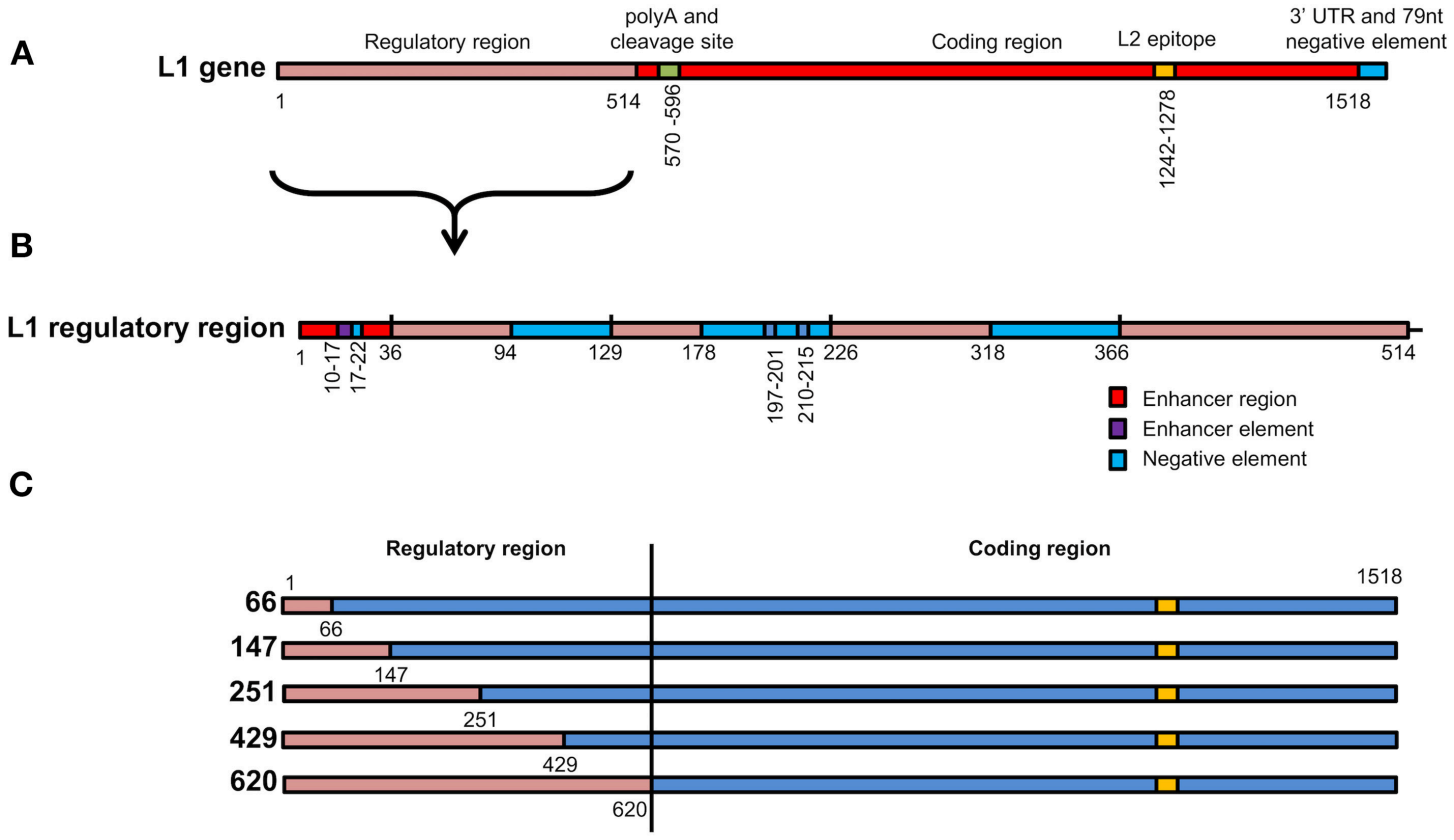
Schematic representation of L1 chimeras **(A)** L1/L2 gene showing various elements found on the gene. The first 514 nucleotides contain elements that regulate gene expression. L2 epitope indicates the replacement of amino acids on the L1 protein with L2 108–120. **(B)** Enlargement of the L1 regulatory region from 1 to 514 showing position of enhancer regions, enhancer elements and negative elements. **(C)** Schematic representation of the 5 *wt/L1/L2* chimeras. Red gene regions are from *wt L1* while blue elements are from the high-GC content L1 construct.

The *L1/L2* chimaeric gene *ChiF/SAF* (Genbank number: AY177679) (Varsani et al., 2003a; McGrath et al., 2013) was used as a starting point for sequence modification (Figure 1A). Sequences were generated using GeneOptimizer® (Life Technologies, USA), a multi-parameter gene optimization software tool which allowed a balance of codon choice (human or tobacco), and of GC or CpG dinucleotide content. Briefly, by using a sliding combination window and applying different emphasis to certain gene optimization parameters (in this case preferred dicot codon usage and a certain GC content), the described algorithm allowed us to identify DNA sequences showing the best balance between a given GC content and a preferred dicot codon choice, as assessed by the software. In the case of the back-translated (BT) *SAF2* sequence, only tobacco-preferred codons were used for back-translating the amino-acid sequence. The resulting sequences were then assembled from oligonucleotides, cloned and sequence verified (GeneArt, Regensburg, Germany) (Table 1).

**Table 1:** Summary of genes with varying GC content.

**Name**	**GC content**	**Details**	**L1/L2_108−120_ Chimera**
*Chi F*	38.27% (WT)	Wild type gene – HPV codon usage	Yes
*SAF 2*	34.64% (BT)	Tobacco codon optimization (TCO)	Yes
*SAF 3*	50.88%	TCO with elevated GC content	Yes
*SAF 4*	61.01%	TCO with elevated GC content	Yes
*SAF 5*	43.06% (CpG)	TCO with elevated CpG dinucleotides	Yes
*SAF*	62.52%	Human codon optimized	Yes
*hL1*	62.20% (hL1)	Human codon optimized *L1* gene	No

To investigate if the increase in mRNA and protein levels was due to removal of negative elements found in the 5′ end of the HPV16 *L1* DNA, 5 chimeras were created where the 5′ end of the gene was replaced with *wt* sequence. In these *wt/L1/L2* chimeras the following sequences were replaced using assembly PCR: 1–66, 1–147, 1–251, 1–429, and 1–620; they were called *66, 147, 251, 429*, and *620* (Figure 1C). The *wt/L1/L2* chimeras were created using *wt L1* as template for the 5′ PCR and the 3′ end was created using human codon-optimized *L1/L2* as template using primers listed in Table 2. The middle primers had overlapping sequencing to allow amplification of whole gene in a second PCR reaction.

**Table 2:** Primers used in plasmid construction.

**Primer name**	**Primer sequence**	**5′ Primer binding site**	**Product size (kb)**
ChiF F	5′-ACTGCAGACGTTATGACATAC-3′	1,170	99
ChiF R	5′-TTCCACTAATGTGCCTCCTG-3′	1,229	
SAF-2 F	5′-ACCAGGTGGAACTCTTGTTG-3′	1,298	97
SAF-2 R	5′-AGTGGATCTTCCTTTGGAGC-3′	1,355	
SAF-3 F	5′-CGATCTCCAGTTCATCTTCC-3′	1,181	100
SAF3 R	5′-TTCCAATCCTCAAGGATAGTG-3′	1,240	
SAF-4 F	5′-AAGAGTACGACCTCCAGTTC-3′	1,195	96
SAF-4 R	5′-AAGGATGGTGGAGTTCATGG-3′	1,251	
SAF-5 F	5′-TACTCTTCAAGCTAATAAGTCC-3′	727	105
SAF-5 R	5′-AAGAATCGCCGTATGGTTCG-3′	790	
SAF F	5′-ACTTCAAGGAGTACCTGAGG-3′	1,152	103
SAF R	5′-TGTGGATGTAGGTCATCACG-3′	1,215	
SAF F66	5′-AGTATCTAAGGTTGTAAGCACCGATGAGTACGTGG-3′		
Chi F R66	5′-ACGTACTCATCGGTGCTTACAACCTTAGATACTGG-3′		
SAF F147	5′-AGTTGGACATCCCTATTTCCCCATCAAGAAGC-3′		
ChiFR147	5′-CTTCTTGATGGGGAAATAGGGATGTCCAACTGC-3′		
SAF F251	5′-TGACCCCAATAAGTTTGGCTTCCCCGACACCAGC-3′		
ChiF R251	5′-TGGTGTCGGGGAAGCCAAACTTATTGGGGTCAGG-3′		
SAF F429	5′-CAGGTGTGGATAACAGAGAATGCATCAG-3′		
ChiF R429	5′-CTGATGCATTCTCTGTTATCCACACCTGCATTTGC-3′		
SAF F620	5′-GTTGATACTGGCTTTGGTGCCATGGACTTCACCACC-3′		
ChiF R 620	5′-GTGGTGAAGTCCATGGCACCAAAGCCAGTATCAACC-3′		

### Subcloning of genes into plant expression vectors

Two binary *Agrobacterium* non-replicative plant expression vectors were used to compare HPV chimera expression: these were pTRAc, which targets the expressed protein to the cytoplasm, and pTRAkc-rbcs1-cTP which targets the protein to the stroma in chloroplasts via the chloroplast-transit peptide sequence of the potato *rbcS1* gene (where *rbcS1* is the ribulose bisphosphate carboxylase small chain 1) (vectors kindly provided by Prof. Rainer Fischer, Fraunhofer Institute for Molecular Biology and Applied Ecology, Germany) (Maclean et al., 2007). The genes were excised with 5′ *Bsp*HI or *Mlu*I and 3′ *Xho*I and directionally cloned into *Afl*III, *Xho*I sites in pTRAc or *Mlu*I and *Xho*I in the chloroplast targeting vector.

DH5-α chemically competent *E. coli* cells (E.cloni™, Lucigen, USA) were transformed with the plasmid constructs and recombinants selected on ampicillin plates (100 μg/mL). Recombinant clones were screened by colony PCR, using pTRA vector-specific primers (Fwd pTRAc Primer 5′-CATTTCATTTGGAGAGGACACG-3′ and RVS pTRAc Primer 5′-GAACTACTCACACATTATTCTGG-3′) and recombinant genes were verified by pyrosequencing.

### *Agrobacterium*-mediated transient expression

*Agrobacterium*-mediated protein expression was performed as described by Maclean et al. (2007). Electrocompetent *A. tumefaciens* GV3101::pMP90RK were transformed with pTRA constructs, plated on 50 μg/mL carbenicillin, 50 μg/mL rifampicin, and 30 μg/mL kanamycin plates and successful transformation confirmed by colony PCR. Chimeras were co-expressed with or without *Agrobacterium* LBA4404 (pBIN-NSs) containing the *NSs* silencing suppressor gene of Tomato spotted wilt virus (TSWV) (Takeda et al., 2002). Recombinant *Agrobacterium* were grown in induction medium and the *Agrobacterium* suspension was either injected- (small scale—a few leaves) or vacuum-infiltrated (large scale—whole plants) into the abaxial air spaces of 6–8 week old *N. benthamiana* leaves. The plants were grown at 22°C under 16/8 h light/dark cycles and samples harvested 1–10 days post-infiltration (dpi).

### Extraction of protein and RNA from plants

To screen leaf tissue for protein expression, five leaf discs (5 mm diameter, ~0.05 g wet plant mass) were ground in liquid nitrogen, and incubated in 650 μl of high-salt phosphate buffer (0.5 M NaCl). The supernatant was clarified by centrifugation for 20 min (13,000 rpm, desktop centrifuge, 4°C) and L1/L2 protein was detected by western blotting. For RNA extractions RNeasy Plant Mini Kit (Qiagen, Netherlands) was used and RNA was extracted as per manufacturer's instructions. Four leaf discs were homogenized in 450 μL extraction buffer, samples were clarified, and eluted into 30 μL. 1/1,000 dilution was used in qRT/PCR.

### ELISA quantification of L1, L1/L2, and *wt/L1/L2* chimera yields

Plant-expressed L1/L2 chimeras were quantified by capture ELISA using a modified polyvinyl alcohol (PVA)-blocking ELISA method (Studentsov et al., 2002), as described in Pineo et al. (2013). Briefly, a 96-well Nunc Maxisorp microtitre plate (Thermo Fisher, USA) was coated with 1:2,000 CamVir1 (Abcam, UK; a mouse anti HPV-16 L1 MAb) overnight at 4°C, washed and blocked with PVA buffer. Diluted plant cell extract was added to the wells and incubated for 1 h at 37°C, followed by a washing step and the addition of rabbit anti-HPV-16 polyclonal serum (1:1,000) overnight at 4°C. After washing, swine anti-rabbit horseradish peroxidase (HRP) conjugate (1:5,000; DAKO, Denmark) was added to wells, plates were incubation for 30 min at 37°C and the proteins were detected with OPD substrate (DAKO). Plates were developed in the dark, the reaction was stopped with 0.5M H_2_SO_4_ and the absorbance was detected at 490 nm. Total soluble protein (TSP) was determined using a Lowry protein assay (BioRad, USA) as per the manufacturer's instructions, with a bovine plasma IgG standard (BioRad, USA) to normalize the ELISA data.

### Protein detection and quantitation by western blot

Samples were incubated at 95°C for 5 min in loading buffer, separated on 10% SDS-PAGE, then either stained with Coomassie blue or transferred onto a nitrocellulose membrane using the Trans-Blot® SD Semi-Dry Transfer Cell (Bio-rad, USA) for western blot analysis. L1 proteins were detected CamVir1 (1:10,000; Abcam, UK), a monoclonal antibody against HPV16 (http://www.abcam.com/hpv16-l1-antibody-camvir-1-ab69.html). MAbs were detected with secondary goat-anti-mouse-alkaline phosphatase conjugate (1:10,000; Sigma Aldrich, USA) and blots developed with NBT/BCIP substrate (Roche, Switzerland). Proteins were measured by semiquantitative analysis by measuring the density of the band on a western blot or Coomassie stained bands in comparison to a known protein concentration standard and purified L1 that had previously been quantitated (Pineo et al., 2013), using GeneTools software (SYNGENE, UK) on scanned images.

### Quantitative RT-PCR

RT-PCR reactions were performed on RNA extracted from leaves using a SensiMix One-Step real-time RT-PCR kit (Quantace, UK) and a Rotor-Gene RG-3000A real-time PCR machine (Qiagen). As a positive control L1 RNA was *in vitro*-transcribed using Ribomax™ Large Scale RNA Production System—T7 kit (Promega, USA). RT-PCR reactions (25 μL) contained *in vitro*-transcribed L1 RNA or 10 μL of the RNA extracted from leaves, 50 mM MgCl_2_ and 50 pmol of forward and reverse primers to amplify a 100 bp DNA fragment. The forward and reverse primer sequences are given in Table 2. The reaction profile used was as follows: 49°C, 30 min; 95°C, 10 min; 45 cycles of 95°C, 15 s, 54°C, 15 s, and 72°C, 15 s. All real-time RT-PCR data was analyzed using the Rotor-gene 6, Version 6.0 (Build 27) software (Corbett Research).

## Results

### Modification of L1/L2 chimera GC content

To determine the impact of GC content on protein expression and mRNA stability, HPV 16 L1/L2_108−120_ chimera coding was changed to give genes with varying GC contents (Table 1). The *wt ChiF* has 38% GC content and the native HPV codon usage was preserved; in the *SAF2* back-translated gene the GC content was kept similar to the *wt* type gene, but tobacco (*N. benthamiana*) preferred codons were used. In *SAF3* and *SAF4* the GC content was 50 and 60% respectively, and the codons used were the ones preferred by tobacco plants. *SAF* was made using human codon usage, with 63% GC content. Additionally, *SAF5* was made with 43% GC content and elevated CpG dinucleotide content with the idea of determining the possible effect of methylation on protein expression. The HPV-16 *L1* gene was human codon optimized for a gene with 62% GC content (*hL1*).

To investigate whether the change in mRNA and protein levels was due to negative elements found in the 5′ end of the HPV-16 *L1* DNA (Figure 1B), 5 chimeras were created from *SAF* with the first 66, 147, 251, 429, or 620 5′ nucleotides replaced with *wt L1* sequence from *ChiF*; chimeras were called *wt/L1/L2*. Schematic design of the 5 *wt/L1/L2* chimeras is shown in Figure 1C. These genes were also expressed in plants and mRNA and protein levels measured.

### Comparative expression of *hL1* and the *L1/L2* chimaeric genes in plants

**Figure 2:**
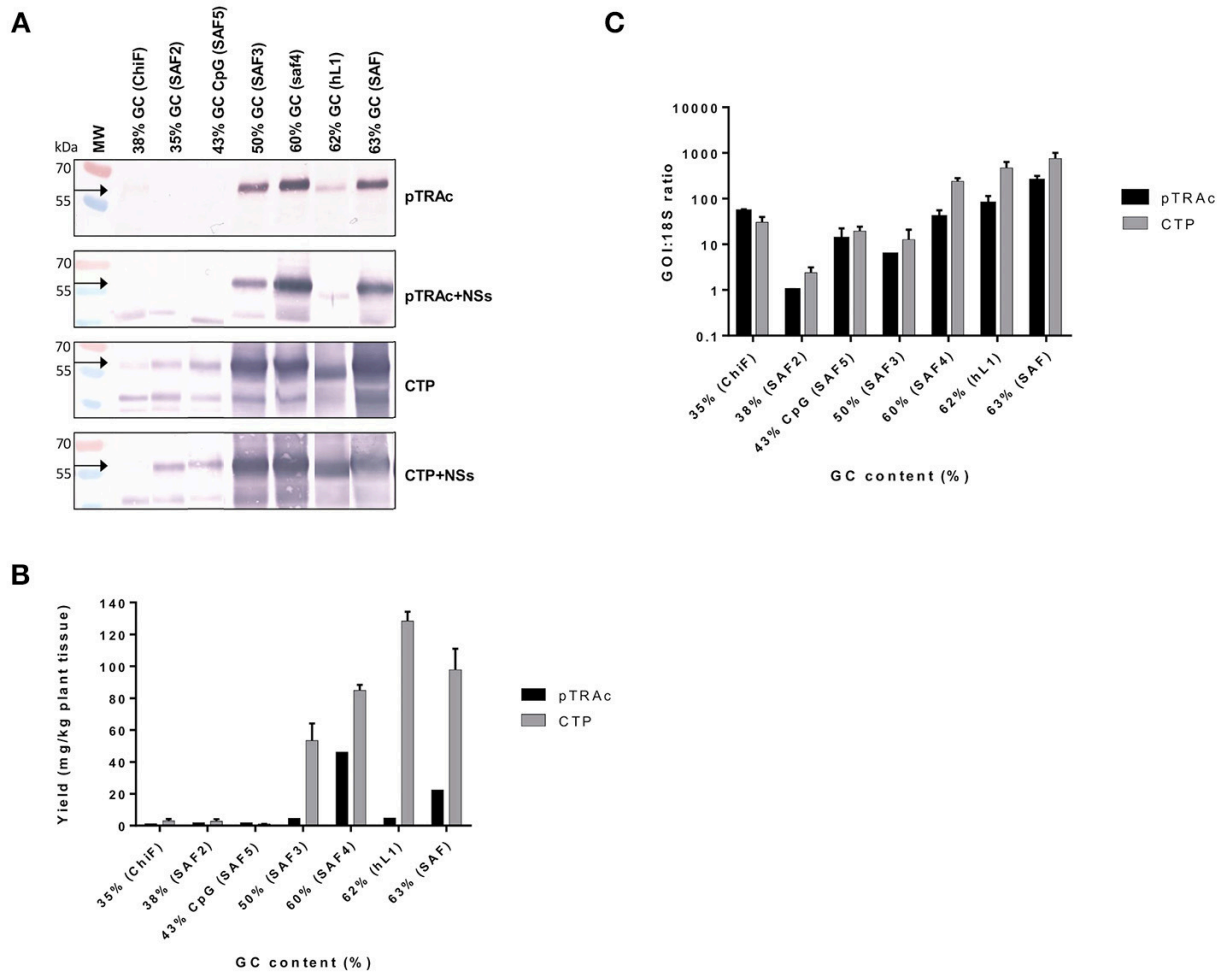
Comparative expression of L1/L2 and hL1 with different GC contents using pTRAc and pTRAkc rbcs1-CTP (CTP). Protein was expressed with or without NSs and extracted 5dpi. **(A)** Western blot detection of L1/L2 and hL1. MW, molecular weight marker, arrow indicates position of L1 and L1/L2 proteins. Equal volumes of sample were loaded. **(B)** Protein yield as mg/kg fresh weight determined by capture ELISA. Results represent 3 biological repeats, error bars indicate ±SEM. **(C)** Comparison of RNA transcription levels of *L1* and chimeras targeted to different subcellular locations. mRNA levels were measured as ratio of 18S RNA to normalize for extraction variation.

L1/L2 chimeras and hL1 were transiently expressed in 8-week old *N. benthamiana* plants and proteins were extracted at 3, 5, 7, and 10 dpi. Accumulation levels of L1/L2 and hL1 protein targeted to the cytoplasm or chloroplast with or without co-expression of the silencing suppressor NSs, were compared by western blots at 5 dpi (Figure 2A) and quantitative ELISA (Figure 2B). Clear bands in western blots below the expected L1 chimera size of 55 kDa are almost certainly products of proteolysis, which we have reported previously for plant-produced HPV-16 and HPV-11 L1s (Varsani et al., 2003b; Kohl et al., 2007). All experiments were repeated at least 3 times and ELISA results are presented as mean averages. Co-infiltration of plants with NSs did not enhance protein accumulation at 5 dpi. Targeting the protein to the chloroplast increased protein accumulation levels 3 to 10-fold relative to cytoplasmic levels (Figure 2B). Higher accumulation levels were seen for all the chimeras with GC contents of 50% and above when targeted either to the cytoplasm or to the chloroplast. The yield of L1/L2 protein ranged from 1.4 mg/kg fresh weight for ChiF to 128 mg/kg for hL1 when targeted to the chloroplast, whereas expression levels in the cytoplasm were 1.2 and 4.8 mg/kg for ChiF and hL1 respectively. L1 accumulated to between 0.1 and 0.9% of TSP.

Protein expression levels also varied with the GC content of the chimeras: the *wt ChiF* and the back-translated *SAF2*, which had GC contents of 38 and 35% respectively, hardly expressed at all. The *SAF5* gene with 43% GC content and high levels of CpG was also minimally expressed. *SAF3* with 50% GC content produced L1/L2 levels up to 53 mg/kg; *SAF4* with 60% GC content expressed up to 85 mg/kg. The two genes that used mammalian codon preference had the highest accumulation levels: *SAF* with 63% GC content expressed protein up to 100 mg/kg and *hL1*—HPV-16 L1 with a GC content of 62%—expressed the highest level of any construct, with up to 128 mg/kg (Figure 2B).

### Comparative mRNA levels in plants

To investigate whether the difference in protein expression levels were due to translation or transcription, as it has been postulated that the RNA secondary structure might have an effect on translation and RNA half-life, we used the ratio of specific mRNA levels to plant 18S RNA as determined by quantitative RT-PCR to normalize for extraction variation. Addition of the NSs did not increase mRNA levels (data not shown), while expression from constructs targeting the protein to the chloroplast increased mRNA levels 2.5–8.8 times compared to targeting to the cytoplasm (Figure 2C). This increase could possibly come from changes in secondary structure of the mRNA with the chloroplast targeting peptide. In plants infiltrated with the *L1/L2* the genes with GC content of 60% and above, targeting the resulting protein to the chloroplast showed a 5 to 100-fold increase in mRNA levels. Interestingly both the back-translated *SAF2* gene (35% GC) and *SAF5* with the high CpG level expressed higher mRNA levels than *SAF3* with 50% GC content. The latter produced low mRNA levels, but protein levels were high when targeted to the chloroplast. This could confirm the theory that the protein is protected from degradation by targeting it to the chloroplast. Overall the mRNA levels did not show the same pattern as protein accumulation.

### Creation and comparative mRNA and protein levels of *wt/L1/L2* chimeras in plants

**Figure 3:**
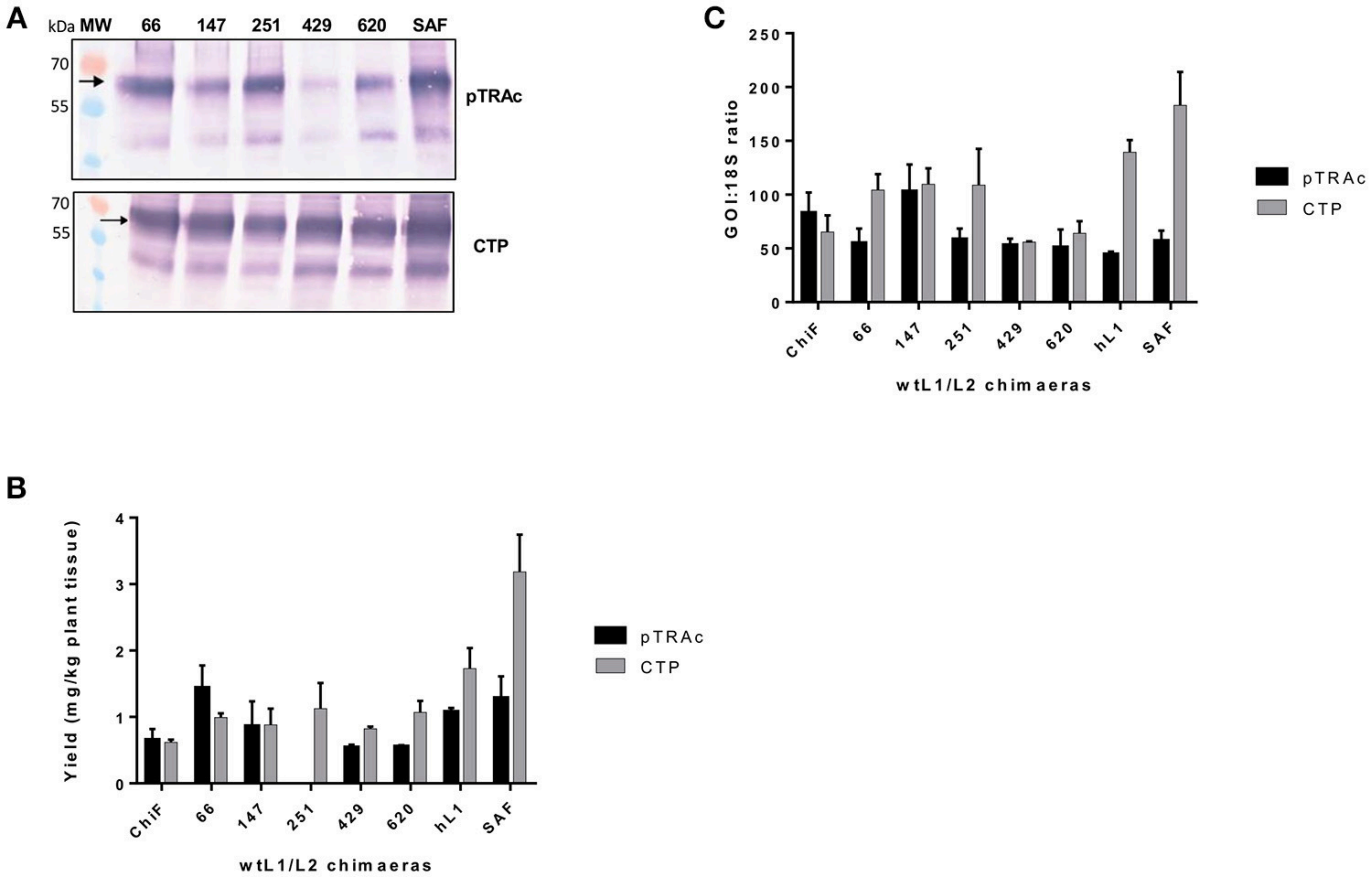
Comparative expression of *wt/L1/L2* with L1/L2 and L1 using pTRAc and pTRAkc rbcs1-CTP (CTP). **(A)** Western blot detection of *wt/L1/L2*, L1/L2, and L1. MW, molecular weight marker, arrow indicates position of L1, L1/L2, and *wt/L1/L2* proteins. Equal volumes of sample were loaded. **(B)** Protein yield as mg/kg fresh weight determined by capture ELISA. Results represent 3 biological repeats, error bars indicate ±SEM. **(C)** Comparison of RNA transcription levels of *L1* and chimeras targeted to different subcellular locations. mRNA levels were measured as ratio of 18S RNA to normalize for extraction variation.

Previous studies have shown that negative elements in the 5′ end of HPV-16 *L1* DNA were responsible for reduced expression of the gene in mammalian and insect cells (Figure 1B). To investigate the influence of these negative elements on protein expression, 5 chimeras were created from *SAF* with the first 66, 147, 251, 429, or 620 5′ nucleotides being replaced with *wt L1* sequence from *ChiF*; chimeras were called *wt/L1/L2*. Schematic design of the 5 *wt/L1/L2* chimeras is shown in Figure 1C. These genes were also expressed in plants and mRNA and protein levels measured. Protein accumulation was assessed by western blot and ELISA (Figures 3A,B). Interestingly, when the proteins were targeted to the chloroplast, the variation in *wt/L1/L2* accumulation was similar for the first three *wt/L1/L2* chimeras (66, 147, and 251). When *wt/L1/L2* was targeted to the cytoplasm, however, constructs 429 and 620 showed markedly less protein accumulation, which is evident in the western blot and ELISA (Figures 3A,B). In summary, there was not a profound effect on protein accumulation by replacing the 5′ regulatory regions of the *L1* gene with *wt* sequence but replacing the protein from nt 429 onwards results in decrease of expression.

Comparing the wildtype ChiF mRNA levels to the mRNA levels of *wt/L1/L2* chimeras, it was interesting to note that the two chimeras 429 and 620 had similar mRNA levels when proteins were targeted to the chloroplast and cytoplasm (Figure 3C). Overall mRNA levels were 1.6 times lower for these two chimeras and protein expression was also lower than all the other chimeras again showing a region of the L1 that has an influence on protein expression levels.

## Discussion

In previous work we and others determined that using human codon optimization—which increased the GC content of the HPV L1 gene to above 60%—increased HPV-16 L1 protein expression in plants (Biemelt et al., 2003; Maclean et al., 2007). Here we wished to systematically explore the effect of GC content on gene expression in plants by creating genes encoding the same HPV L1/L2 chimera with 6 different GC contents. Additionally, as there is an enormous body of work that describes that negative elements in the L1 gene affect protein expression in the natural cycle of the virus (Schwartz, 1998; Collier et al., 2002; Rollman et al., 2004; Zhao and Schwartz, 2008), we further wished to investigate if the HPV L1 negative elements also function in plants to limit L1 expression.

The *wt ChiF* has 38% GC content and the native HPV codon usage was preserved. If the most preferred dicot codons are used to back translate any given gene this results in sequence with a very low GC content, usually significantly below 45%. *SAF2* is the back-translated gene where tobacco (*N. benthamiana*) preferred codons were used, resulting in a gene with 35% GC content. In order to be able to balance the use of preferred dicot codons and step by step increasing the GC content of the resulting DNA sequence, the multi-parameter gene optimization approach described in detail by Raab et al. was used (Raab et al., 2010). By using this sliding combination window and applying different emphasis to certain gene optimization parameters (in this case preferred dicot codon usage and a certain GC content), optimal DNA sequences showing the best balance between a given GC content and a preferred dicot codon choice were created. *SAF3* and *SAF4* were created in this way, resulting in genes with GC content of 50% and 60% respectively, but using codons preferred by tobacco plants. The positive control genes *SAF* and HPV-16 L1 (*hL1*) were made using human codon usage, resulting in genes with 63 and 62% GC content, respectively.

Lastly, *SAF5* was created which had a 43% GC content and elevated CpG dinucleotide content. This was done as previously researchers have shown that aggregation of CpG dinucleotides positively influenced expression in mammalian cells. We therefore speculated that a similar mechanism might positively increase expression in plants as well (Bauer et al., 2010; Krinner et al., 2014). While this was plausible as a hypothesis, the resolution of the experimental approach was not sufficient to infer a putative effect of CpG islands.

### Transient expression of hL1, L1/L2, and *wt/L1/L2* in plants

In the present study we confirmed our previous results that L1-based protein accumulation was enhanced when the protein was targeted to the chloroplast: this was true for the L1 protein, and the L1/L2 chimera. We further determined that human codon-optimized genes with the highest GC content showed the highest expression levels. A plant codon-optimized gene with a similar GC content of 60% showed approximately 66% of the expression level of the hL1. In previous codon-optimization studies on papillomavirus gene expression in mammalian cells, L1, E5, and E7 genes all showed increased mRNA translation efficiency and thus higher protein expression when human codon-optimized (Leder et al., 2001; Liu et al., 2002; Disbrow et al., 2003). When a HPV-16 L1-E7 hybrid gene was created for DNA vaccination, there was increased expression and thus immune response when the gene was human codon-optimized (Cheung et al., 2004). Further, Collier et al. (2002) created a mutant L1 cDNA which was codon-optimized to increase the GC content of the AU-rich HPV-16 genome: they demonstrated high levels of both L1 mRNA and protein (Collier et al., 2002).

In this study we also measured mRNA levels in plants infiltrated with the genes with varied GC content. We observed a 170-fold increase in mRNA levels when comparing the *wt* gene to the human codon-optimized genes. The *SAF3* plant codon-optimized gene with GC content of 50% showed only a 10-fold increase in mRNA levels; the *SAF4* gene with 60% GC content showed a 50-fold increase (Figure 2C). Protein expression levels also increased with increasing GC content (Figures 2A,B). This confirms that changing the codon composition of *L1* from an AT-rich to GC-rich results in increase of accumulation of L1 mRNA, and that the effect on protein expression is thus mainly at the transcriptional level. Interestingly, we also found an increase in mRNA in the proteins targeted to the chloroplast. This was a bit surprising as it is thought that targeting the protein to the chloroplast could potentially increase accumulation by sequestering the protein away and thereby protecting it from degradation (Maclean et al., 2007). Our results indicate that the mRNA of the chloroplast targeted proteins is more stable, which might be inadvertently due to the addition of the signal sequence coding region giving the mRNA more stability.

The increase in mRNA levels may be due to increase in L1 mRNA half-life (Mori et al., 2006); additionally, poor expression of the *wt* gene could be due to the presence of inhibitory RNA elements. *L1* late gene expression is strongly influenced by post-transcriptional gene regulation (Zheng and Baker, 2006); therefore viral elements and cellular RNA binding factors are important in regulating the HPV-16 genes (Mole et al., 2006). It is thought that codon-optimization affects RNA inhibitory sequences and thus influences the relevant binding protein interactions. Therefore, it could be argued that the increased translation efficiency is solely due to disruption of these protein binding sites on the mRNAs with little effect actually coming from the codon optimization (Zhao and Schwartz, 2008).

### Mapping of negative elements on *L1* gene

The first 514 nucleotides (nt) of the *wt L1* coding region contain multiple RNA sequences that inhibit gene expression (Figures 1A,B). These sequences reduce mRNA levels as well as inhibiting translation. Some sequences are thought to be splicing silencers elements and binding of heterogeneous nuclear ribonucleoprotein (hnRNP) A1 to these sites inhibits transcription (Schwartz, 2013). As we wanted to investigate if gene expression in plants was enhanced due to change in some of the negative elements described, we created 5 *wt/L1/L2* chimeras where we changed the 5′ end of the human codon optimized *L1/L2* gene back to the *wt* sequence (Figure 1C). Changing the first 147 nt or more in the *L1* gene back to its *wt* sequence decreased protein accumulation 2-fold (Figure 3A). Overall, we found that changing small parts of the 5′ end of the *L1* gene decreased protein accumulation in plants. This suggests that these negative sequences on the L1 gene may also be effective in plants. This was surprising as the L1 5′ and 3′ UTR regions dramatically influence expression levels, but as the 5′ and 3′ UTR were unchanged in our constructs, there are probably elements on L1 influencing expression levels and these are also effective in plant cells. While additional experiments designed to determine whether increased translation efficiency is solely due to disruption of negative regulatory protein binding sites on the mRNAs by codon optimization would be useful, unfortunately this did not fall into the scope of this work.

To determine if post-transcriptional gene silencing played a role in L1 mRNA levels and thus the expressed protein levels, in all experiments the silencing suppressor protein NSs from the TSWV was expressed together with the L1 proteins. NSs is thought to interfere with steps that generate the dsRNA, thereby inhibiting silencing (Takeda et al., 2002). However, we did not observe an increase in protein or mRNA levels, indicating that gene silencing did not play a role in controlling protein expression in this system. In a recent study by Jackson et al., codon optimization, RNA instability motifs, blocking of sRNA binding sites and randomization of non-rare codons were investigated to provide rules for efficient transgene expression in plants and reduce gene silencing (Jackson et al., 2014). The authors showed that eliminated on such motifs yielded up to 935-fold increase in gene expression. Therefore, these additional rules could be used to increase gene expression of HPV-L1 in plants.

Overall, we determined that constructs based on the main HPV-16 capsid protein gene *L1* were not well expressed in plants if the *wt* nucleotide sequence was used. We showed that mRNA levels increased 10-fold when the GC content was increased from 35 to 43% or higher, but protein expression only increased once the GC content was above 50%. Changing the first 620 nt on the 5′ end of *L1* back to *wt* sequence showed a 50% reduction of protein expression. This indicates that the negative elements found in the 5′ end of the *L1* gene do play a significant role in L1 protein expression. We were not able to elucidate the effect of each element, but determined that this region plays an important role in protein expression in plants. While it is clear that in this study changing codon usage quite dramatically affected expression levels of our chosen gene, it cannot be claimed that this is the only reason for the differences. For instance, mRNA splicing and nuclear export were not studied, and therefore we cannot generally exclude that aberrant splicing, inefficient transcript processing or nuclear export also affected the results.

This work shows that optimization of protein expression in plants remains a challenge, but that the challenge is surmountable. In general, our research group has found that GC content above 50% is necessary for adequate protein expression of HPV and other proteins, such as H5N1 influenza virus haemagglutinin, in plants (Maclean et al., 2007; Pereira et al., 2009; Mortimer et al., 2012; Pineo et al., 2013). Our findings are also of significant interest in the continuing search for a second generation of affordable HPV vaccines.

## Author contributions

IH and AC wrote the manuscript. MW performed the experiments, AC compiled figures. MG designed and synthesized the genes. IH and ER designed and conceptualized the study.

### Conflict of interest statement

MG declares a competing financial interest: Geneart AG performs gene design optimization as a free service with the genes that it sells. The remaining authors declare that the research was conducted in the absence of any commercial or financial relationships that could be construed as a potential conflict of interest.
